# Supplementary material for: Impact of rotavirus vaccination in Malawi from 2012 to 2022 compared to model predictions before, during, and after the COVID-19 pandemic
Source: medRxiv. 2024 May 31:2024.05.29.24308124. Preprint. [Version 1] doi: 10.1101/2024.05.29.24308124 (PMC11160830; doi:10.1101/2024.05.29.24308124)
Supplement: 1 [file NIHPP2024.05.29.24308124V1-supplement-1.pdf]

medRxiv preprint doi: https://doi.org/10.1101/2024.05.29.24308124; this version posted May 31, 2024. The copyright holder for this preprint (which was not certified by peer review) is the author/funder, who has granted medRxiv a license to display the preprint in perpetuity.
It is made available under a CC-BY 4.0 International license .

Table S1. Model parameters.

**Fixed parameters**	**Variable**	**Value**	**Source**
Birth rate	$B(t)$	0.0366 to 0.0550 year^{-1}	[39]
Duration maternal immunity	$1/\omega_M$	26 weeks	[40]
Duration of infectiousness			
First infection	$1/\gamma_1$	1 week	[41]
Subsequent infections	$1/\gamma_2$	0.5 week	[42,43]
Duration of temporary immunity	$1/\omega$	13 weeks	[44], assumption
Relative risk of reinfection			
Following first infection	σ_1	0.62	[45,46]
Following second infection	σ_2	0.35	[45,46]
Relative infectiousness			
Following first infection	ρ_1	0.5	[45,46]
Following second infection	ρ_2	0.1	[47]
**Estimated parameters**	**Variable**	**Maximum a posteriori estimate (95% credible interval)**	
Basic reproductive number	$R_0 = \beta_0/\gamma_1$	78.8 (70.5-96.2)	[12]
Reporting rate (mean)	h	0.017 (0.016-0.018)	[12]
Amplitude of seasonality in transmission	b	0.174 (0.113-0.294)	[12]
Phase shift of seasonal transmission	ϕ	6.9 (4.0-11.2) weeks	[12]

Table S2. Vaccine parameters. Models 1 and 2 assume no waning of vaccine-induced immunity; the probability of responding to each vaccine dose was estimated from seroconversion data from the RV1 vaccine trial in Malawi [8]. Vaccine-related parameters for Models 3 and 4 were estimated by fitting to the post-vaccination time series of RVGE cases at Queen Elizabeth Central Hospital through August 2017 [12].

	**Probability of responding to first vaccine dose**		**Probability of responding to second dose among those responding to the first dose**		**Duration of vaccine-induced immunity (years)**	
	**Mean**	**Distribution**	**Mean**	**Distribution**	**Mean**	**Distribution**
Model 1	0.687	Beta(24.7,11.3)	0.687	Beta(24.7,11.3)	--	--
Model 2	0.527	Beta(19,17)	0.895	Beta(32.2,3.8)	--	--
	**Mean**	**95% CrI**	**Mean**	**95% CrI**	**Mean**	**95% CrI**
Model 3	0.619	(0.471, 0.750)	0.604	(0.467, 0.751)	0.645	(0.526, 0.794)
Model 4	0.483	(0.375, 0.605)	0.791	(0.655, 0.913)	0.965	(0.617, 1.644)

medRxiv preprint doi: https://doi.org/10.1101/2024.05.29.24308124; this version posted May 31, 2024. The copyright holder for this preprint (which was not certified by peer review) is the author/funder, who has granted medRxiv a license to display the preprint in perpetuity.
It is made available under a CC-BY 4.0 International license .

Table S3. Overall vaccine effectiveness estimates by year. The overall effectiveness (OE) is calculated based on the observed number of RVGE cases compared to the model-predicted incidence of RVGE with no vaccination in each year following vaccine introduction.

	Age group			
Year	All ages <5 years old	<1 year old	1-<2 years old	2-<5 years old
2012*	2.7%	11.1%	-44.7%	-49.1%
2013	33.3%	48.5%	-19.5%	-320.5%
2014	55.5%	66.0%	22.7%	-68.4%
2015	26.6%	48.1%	-37.1%	-387.3%
2016	16.6%	36.4%	-55.6%	-444.4%
2017	18.8%	34.4%	-11.5%	-1150.2%
2018	11.2%	37.1%	-70.7%	-741.9%
2019	16.7%	37.7%	-55.0%	-610.0%
2020#	-15.0%	39.9%	-214.4%	-1134.4%
2021	17.6%	40.9%	-55.7%	-562.2%
2022$	1.4%	24.0%	-100.2%	100.0%
**2013-2022$**	**36.0%**	**52.5%**	**-18.6%**	**-336.4%**

*Rotarix vaccine was introduced on October 29, 2012.
[#] Excludes the period from April 5 to October 10 when surveillance was halted because of the COVID-19 pandemic.
[$] Limited to data through June 25, 2022.

Table S4. Model-predicted overall vaccine effectiveness. The overall effectiveness (OE) predicted by each of the four vaccination models compared to the model-predicted incidence of RVGE with no vaccination is presented for the best-fit models.

	All ages <5 years old	<1 year old	1-<2 year old	2-<5 years old
Model 1	58.9%	60.8%	52.5%	19.5%
Model 2	41.1%	43.0%	34.3%	10.4%
Model 3	21.2%	30.1%	-5.3%	-258.5%
Model 4	22.1%	29.3%	2.3%	-247.2%

medRxiv preprint doi: https://doi.org/10.1101/2024.05.29.24308124; this version posted May 31, 2024. The copyright holder for this preprint (which was not certified by peer review) is the author/funder, who has granted medRxiv a license to display the preprint in perpetuity.
It is made available under a CC-BY 4.0 International license .

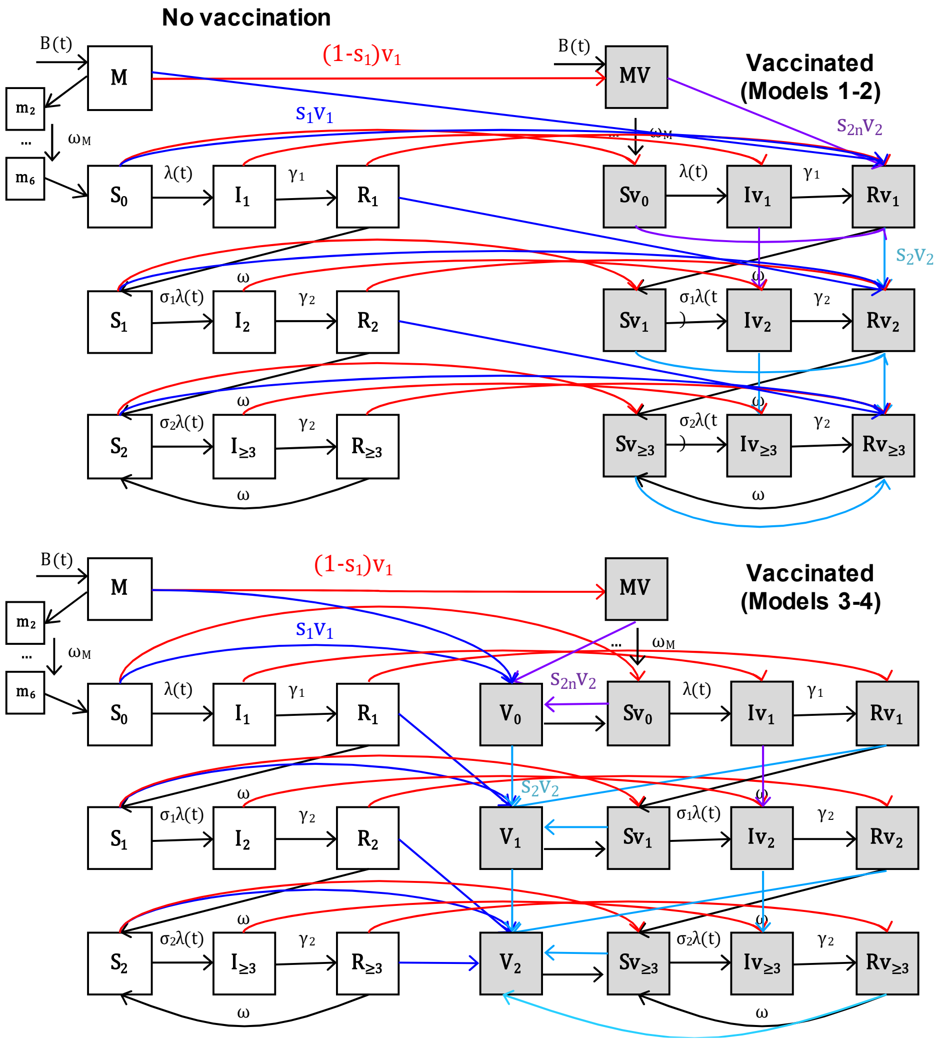

Figure S1. Compartmental diagram of transmission models. Boxes represent the various model states (unvaccinated in white, vaccinated in grey), while the lines represent the movements between model states for (top) Models 1 and 2 (assuming vaccine-induced immunity is comparable to immunity from natural infection) and (bottom) Models 3 and 4 (assuming waning of vaccine-induced immunity). The blue and turquoise lines represent the movement of individuals who respond to the first and subsequent doses of rotavirus vaccine, respectively, while the red lines represent the movement of individuals who fail to respond to the first dose. The purple lines represent the probability of responding to the second dose among those who failed to respond to the first dose when we assume heterogeneity in vaccine response (Models 2 and 4). Individuals who fail to respond to subsequent doses remain in their respective vaccinated compartments. Adapted from [12].

medRxiv preprint doi: https://doi.org/10.1101/2024.05.29.24308124; this version posted May 31, 2024. The copyright holder for this preprint
(which was not certified by peer review) is the author/funder, who has granted medRxiv a license to display the preprint in perpetuity.
It is made available under a CC-BY 4.0 International license .

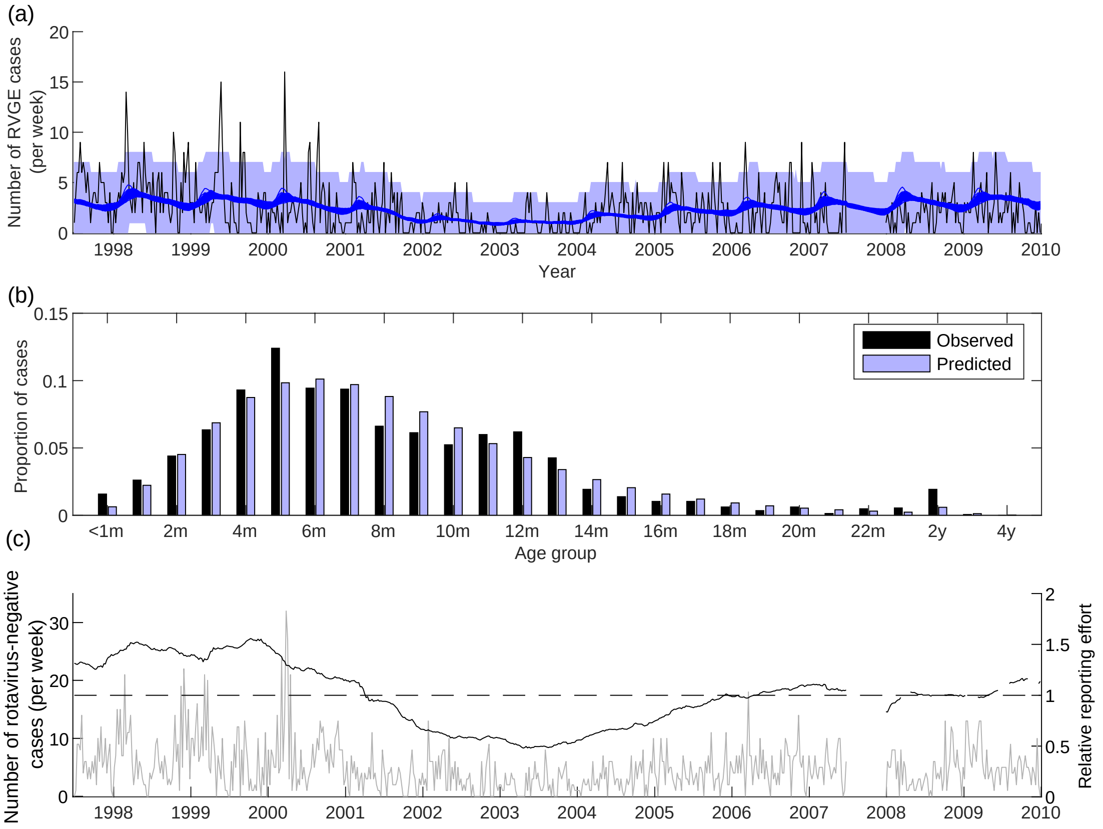

Figure S2. Weekly timeseries of rotavirus-positive and negative cases and fitted model for pre-vaccination period, July 1997-December 2009. (a) The number of rotavirus-associated gastroenteritis (RVGE) cases per week diagnosed at Queen Elizabeth Central Hospital in Blantyre, Malawi is plotted in black, while the fitted models are plotted in blue for 100 samples from the posterior distribution of model parameters. The blue shaded region represents the 95% prediction intervals assuming the observed number of cases per week are Poisson distributed with a mean equal to the model-predicted weekly average number of cases. (b) The age distribution of observed RVGE cases (black bars) is plotted alongside the model-predicted age distribution of RVGE cases (blue bars). (c) The number of rotavirus-negative acute gastroenteritis cases per week are plotted in grey on the left axis, while the relative reporting effort over time is plotted in black on the right axis; the black dashed line shows a relative reporting effort of 1. The reporting effort is calculated from the 105-week (two-year) moving average of the number of rotavirus-negative cases divided by the average for the entire time period.

medRxiv preprint doi: https://doi.org/10.1101/2024.05.29.24308124; this version posted May 31, 2024. The copyright holder for this preprint (which was not certified by peer review) is the author/funder, who has granted medRxiv a license to display the preprint in perpetuity.
It is made available under a CC-BY 4.0 International license .

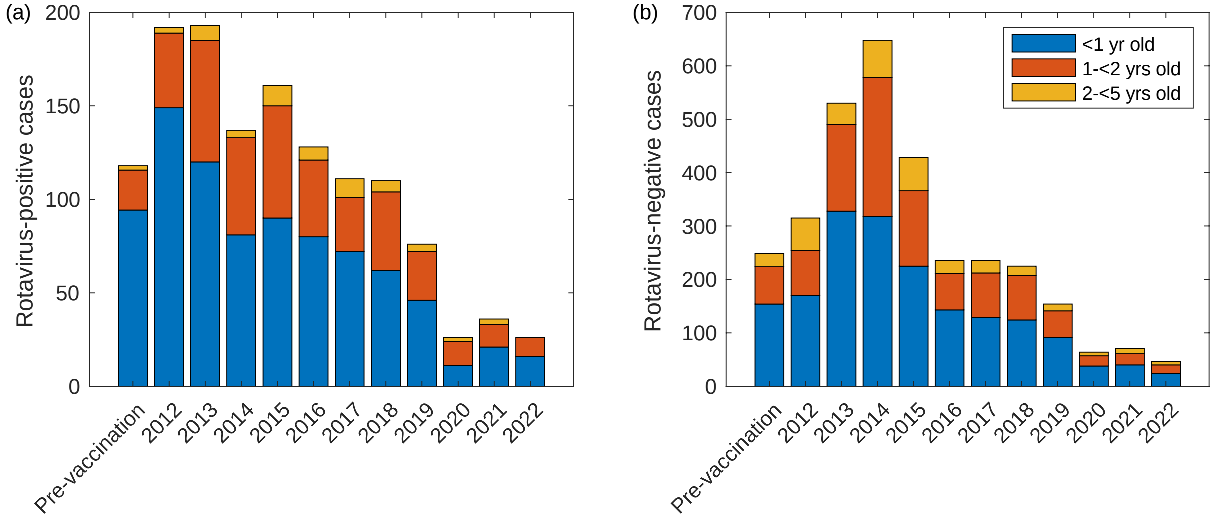

Figure S3. Annual cases of rotavirus-positive and rotavirus-negative acute gastroenteritis by age group, 1997-2022. The number of (a) rotavirus-positive and (b) rotavirus-negative acute gastroenteritis cases presenting to Queen Elizabeth Central Hospital in Blantyre, Malawi are plotted by year and age group (blue <1 year old; red 1-<2 years old; yellow 2-<5 years old). The first bar represents the average annual number of cases from the pre-vaccination period (June 1997-December 2009); data from 1997 and 2007 were excluded because surveillance was only conducted for six months in these years.

medRxiv preprint doi: https://doi.org/10.1101/2024.05.29.24308124; this version posted May 31, 2024. The copyright holder for this preprint
(which was not certified by peer review) is the author/funder, who has granted medRxiv a license to display the preprint in perpetuity.
It is made available under a CC-BY 4.0 International license .

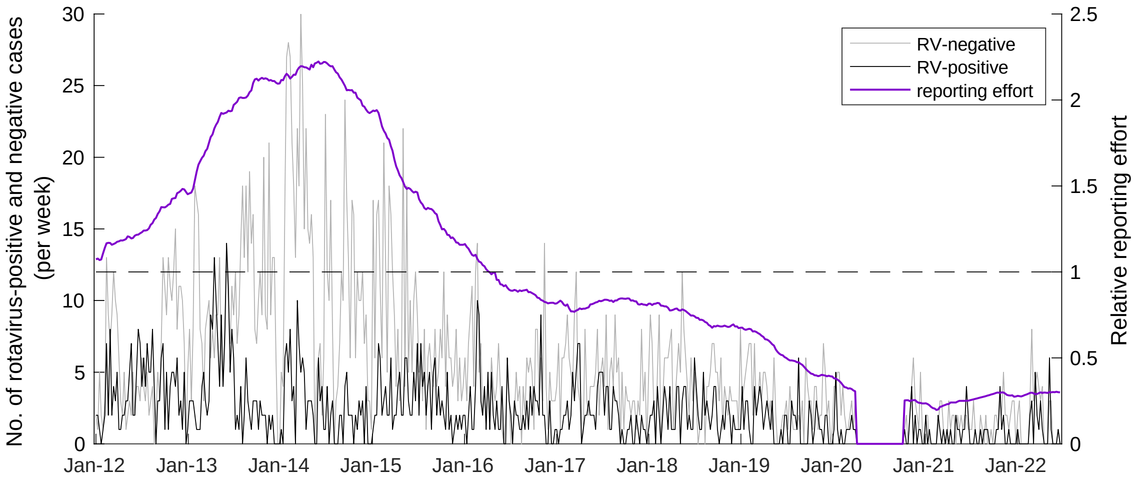

Figure S4. Weekly timeseries of rotavirus-positive and negative cases and relative reporting effort, January 2012-June 2022. The number of rotavirus-positive (black) and rotavirus-negative (grey) acute gastroenteritis cases per week diagnosed at Queen Elizabeth Central Hospital in Blantyre, Malawi are plotted on the left axis. The relative reporting effort over time (purple) is calculated from the 105-week (two-year) moving average of the number of rotavirus-negative cases divided by the average for the entire time period and is plotted on the right axis. The black dashed line shows a relative reporting effort of 1.

medRxiv preprint doi: https://doi.org/10.1101/2024.05.29.24308124; this version posted May 31, 2024. The copyright holder for this preprint (which was not certified by peer review) is the author/funder, who has granted medRxiv a license to display the preprint in perpetuity.
It is made available under a CC-BY 4.0 International license .

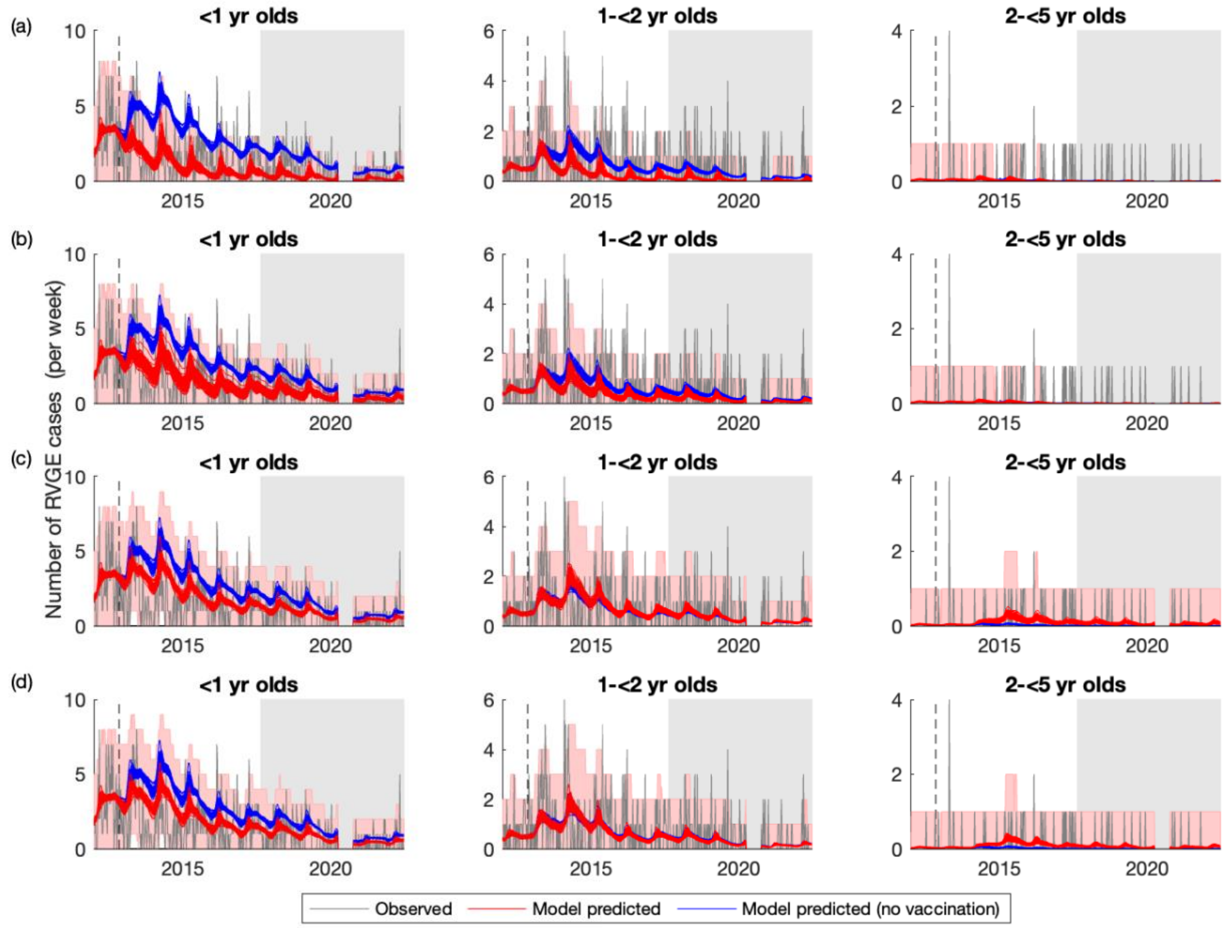

Figure S5. Weekly timeseries of observed and model-predicted rotavirus gastroenteritis cases by age group at Queen Elizabeth Central Hospital, January 2012-June 2022. The observed number of RVGE cases per week for three different age groups (<1 year old, left; 1-<2 year old, middle; 2-<5 year old, right) is plotted in grey, while model predictions for the average weekly number of RVGE cases given current estimates of vaccine coverage (red lines) and assuming no vaccination (blue lines) are plotted for 100 samples from the posterior distribution of model parameters for (a) Model 1, (b) Model 2, (c) Model 3, and (d) Model 4. The red shaded region represents the 95% prediction intervals assuming the observed number of cases per week are Poisson distributed. The dashed vertical line shows the week of vaccine introduction, while the light grey shaded region shows the out-of-sample validation period.
